# FATIGUE OF PALLIATIVE CARE NURSING STAFF AND SELECTED SOCIODEMOGRAPHIC, OCCUPATIONAL AND COGNITIVE PREDICTORS: A CROSS-SECTIONAL STUDY

**DOI:** 10.13075/ijomeh.1896.02520

**Published:** 2025

**Authors:** Karol Czernecki, Grzegorz Nowicki, MichaŁ Graczyk, Barbara Ślusarska

**Affiliations:** 1 “Z Serca Dla Serca” Foundation, Hospice House of Angels for Children, Kępie Zaleszańskie, Poland; 2 Piotrków Academy in Piotrków Trybunalski, Department of Nursing, Piotrków Trybunalski, Poland; 3 Medical University of Lublin, Department of Family and Geriatric Nursing, Faculty of Health Sciences, Lublin, Poland; 4 Nicolaus Copernicus University in Toruń, Collegium Medicum in Bydgoszcz, Department of Palliative Care, Bydgoszcz, Poland

**Keywords:** fatigue, nurses, palliative care, work, demographic, stress

## Abstract

**Objectives:**

The aim of the study is to assess total fatigue levels among nursing staff who provide palliative care services, as well as to identify significant sociodemographic, occupational and cognitive predictors of self-perceived fatigue.

**Material and Methods:**

This cross-sectional study was carried out on a study group of 424 nurses that provide health care services in the palliative care units in Poland. The following scales were employed in the study: *Fatigue Assessment Scale*, *Utrecht Work Engagement Scale*, the *Multidimensional Scale of Perceived Social Support*, *Perceived Stress at Work* and *Professional Quality of Life Scale*.

**Results:**

The average total fatigue level in the study group was 20.78 (SD = 5.41). There was a positive relationship between gender, age, place of residence, marital status, education, perception of social support, occupational stress, and professional quality of life and perceived fatigue. In turn, there was a negative relationship between years of service and perception of social support in the “others” category and perceived fatigue.

**Conclusions:**

The study's results show a significant relationship between perceived fatigue and sociodemographic, occupational and cognitive variables.

## Highlights

- Palliative care nurses experience moderate fatigue.
- Nurses with greater social support outside of family show higher fatigue levels.
- Long-term nurses are less fatigued and are better able to manage workloads.
- Fatigue rises with age and is higher in men, urban tenants, and specialized nurses.

## INTRODUCTION

Fatigue, sometimes referred to by the terms “exhaustion” or “burnout,” is a complex symptom experience that impairs an individual's biological, psychological and cognitive processes. According to Hockey [1], it is a difficult concept to define, but it is understood as being a general feeling of tiredness and weariness [2] that negatively affects many aspects of human life [3]. In recent years, there has been a lot of discussion about the connection between fatigue and occupational health. Occupational fatigue is defined as a multi-cause, general feeling of exhaustion exacerbated by a demanding job allied with insufficient recovery time. In terms of duration, it can be divided into acute and chronic. With regard to its nature, fatigue can be classified into physical, mental or total/global [4]. Acute fatigue is temporary and can be relieved by sufficient rest [5]. Chronic fatigue is a long-term condition caused by a lack of recovery from acute fatigue and experiencing a prolonged exhausting workload, which, most importantly, entails serious health consequences [6]. Physical exhaustion is caused by increased physical work and stress, and it manifests as a loss of energy, power and ability to complete tasks [7]. Mental exhaustion, on the other hand, is caused by extended periods of cognitively demanding work and results in decreased alertness, concentration and impaired ability to perform mental tasks [8]. It should be noted that total fatigue is a distinct type of exhaustion that is defined as a feeling of insufficient energy to complete tasks and can be attributed to prolonged physical and mental fatigue [7].

Global health organizations are focusing on this issue, recognizing it as a significant factor affecting overall well-being and work efficiency. Fatigue is often defined and approached differently, reflecting its multidimensional nature and measurement complexities. The results of the literature review summarizing these issues are shown in Table 1.

**Table 1.** Definitions of fatigue according to key international organizations

Organization	Definition of fatigue
World Health Organization (WHO)	Chronic, profound, disabling and unexplained fatigue, often with coinciding symptoms such as sleep problems or post-exertional malaise [9].
International Labour Organization (ILO)	A state of physical and mental exhaustion that impairs an individual's ability to work effectively and safely, caused by factors such as an excessive workload, a lack of rest, monotonous tasks or long working hours [10].
European Agency for Safety and Health at Work (EU-OSHA)	A major occupational-related health issue caused by prolonged physical or mental exertion, insufficient rest or a lack of sleep. It reduces concentration, decision-making ability and work efficiency, increasing the risk of workplace accidents [11].
National Institute for Occupational Safety and Heath (NIOSH)	A state of exhaustion that impairs physical and mental capacity, frequently caused by long working hours, shift work or insufficient rest. In addition to being a personal health concern, this state poses a serious safety risk in high-risk settings [12].
International Council of Nurses (ICN)	A major issue in the nursing profession, caused by long working hours, staff shortages and the emotional burden of patient care. It causes decreased work efficiency, errors in patient care and professional burnout [13].
Scientific Committee of Occupational Health Nursing (ICOH)	A state that has an adverse impact on nurses’ well-being and professional practice, with cumulative effects such as cognitive functions decline, poor physical health and impaired decision-making abilities [14].

Nursing staff are at risk of fatigue or burnout due to the multi-tasking nature of the work, which includes physical, mental, emotional and organizational demands [15]. Studies have shown that hospital nursing staff experience relatively high levels of exhaustion [16,17], and the COVID-19 pandemic had exacerbated this problem [18]. Research shows that nurses develop higher average levels of exhaustion than doctors, allied health personnel or hospital administrative staff [19]. Nursing staff fatigue leads to a variety of negative outcomes, including health consequences, reduced work efficiency, patient safety and organizational costs [20]. The nurses experiencing higher levels of exhaustion are more likely to experience psychological stress, musculoskeletal disorders or needlestick injuries [21–23]. High nursing staff fatigue is also connected with the risk of driver drowsiness, dissatisfaction with therapeutic decisions, loss of productivity, changes in work schedules, late response time and medication administration errors [23,24]. Nursing staff burnout leads to sickness absence and staff turnover that have financial consequences for the organization [25].

A wide range of factors contribute to nursing staff fatigue. These include high workloads, staff shortages, shift work, increased expectations of patients and their families, insufficient time for professional growth, a decline in leadership, little recovery time, personal factors and organizational culture [26,27]. Other factors include the location of the healthcare facility, the patient-related issues addressed by the nursing staff, the severity of the illness and the prognosis. The provision of palliative care to patients with severe medical condition is a multifaceted task for nursing staff that should anticipate the patients’ and families’ reactions to illness, as well as prepare and support them when dealing with physical, emotional, social, cultural and spiritual crises [28]. One of the hardest and most demanding experiences for nurses is caring for patients at the end of their lives. For this reason, it is of key importance for the palliative care nurses to prioritize their own health and well-being and provide patients with high-quality care during the final stages of their illness [29]. To the best of the authors’ knowledge, no studies have been conducted to describe the level of exhaustion experienced by nursing staff providing palliative care services. Therefore, taking into account the need to understand this phenomenon within the Polish environment, the aim of the study was to assess total fatigue levels among nursing staff that provides palliative care services, as well as to identify significant sociodemographic, occupational and cognitive predictors of self-perceived exhaustion.

## MATERIAL AND METHODS

### Study design

This cross-sectional study was conducted in accordance with recommendations contained in the Strengthening the Reporting of Observational Studies in Epidemiology (STROBE) statement on reporting of observational studies [30].

### Procedure and participants

The study data were collected in the period between June 2023 − August 2023. The data was obtained using 2 methods, namely paper and pencil interview (PAPI) and computer-assisted web interview (CAWI). To collect data using the PAPI method, 456 postal questionnaires were distributed. These were sent to all healthcare facilities in Poland that had contracts with the National Health Fund to provide palliative and hospice care services in 2022 (a total of 228 facilities) [31]. Out of all the questionnaires distributed, 197 were returned, constituting a 43.2% return rate. All questionnaires were correctly completed and could be included in the study. In turn, the social networking site, Facebook, was used to collect CAWI material. In order to obtain data, the administrators of the 7 nursing-focused social media groups with the highest number of likes were requested to share a link to the online survey. The CAWI method was applied to collect 227 correctly completed survey questionnaires.

The participant inclusion criteria included:

– holding the right to practice as a nurse in Poland,
– providing palliative care services,
– giving informed consent to participate in the study.

Before the survey questionnaires were sent by post, a letter outlining its purpose and requesting responses from nurses fulfilling the inclusion criteria was provided. In turn, before beginning the online survey, individuals could become familiar with the purpose of the study and the inclusion criteria on the website. In order to complete the online survey, participants were required to first declare that they fulfilled the inclusion criteria, and answering the posed questions was only possible after providing an affirmative response to the following questions: “Do you meet the above criteria?” (The possible answers were: “yes” or “no.”) According to the STROBE checklist, reporting of observational studies in epidemiology does not require sample size calculation [30].

### Measurements

To achieve the study's goal, a structured questionnaire comprised of 5 standardized scales and a researcher-made tool was utilized. All scales used in the study had acceptable internal consistency.

In order to assess total fatigue or burnout, the authors employed the *Fatigue Assessment Scale* (FAS) by Michielsen et al. [32] in the Polish adaptation of Urbańska [33]. The FAS scale consists of 10 questions addressing various aspects of exhaustion. The questions dealt with both physical and psychological experiences associated with work-related exhaustion. The scale measures not only the total exhaustion level, but also its different manifestations, including low energy, feeling of tiredness, concentration difficulties and sleeping disorders. The scale is intended to collect data on the various aspects of fatigue in order to provide a more complete assessment of this phenomenon. Each question is scored using a 5-point Likert scale, ranging from “I strongly disagree” to “I strongly agree”. The total score on the FAS scale is 10–50. In the study by Michielsen et al. [32], the internal consistency of the FAS was α = 0.90. In this study, Cronbach's α coefficient was α = 0.87, as compared to 0.86 in previous Polish adaptation studies [33]. Work engagement was determined using the *Utrecht Work Engagement Scale* (UWES) by Schaufeli et al. [34].

The study employed a Polish questionnaire available on the author's website [35]. The scale consists of 17 statements on the respondent's work. Each statement is rated using a 7-point Likert scale, starting from 0 − “ never” to 6 − “always”. For the purposes of this study, the total score obtained on the scale was used to calculate total work engagement, where higher scores indicated greater engagement [36]. Due to the inconsistent results of the scale's psychometric analysis in Polish [37,38], the scale was modified for this study, resulting in a high Cronbach's α value of 0.939 [39].

So as to assess perceived social support, the *Multidimensional Scale of Perceived Social Support* (MSPSS) tool by Zimet et al. [40] in the Polish adaptation by Buszman and Przybyła-Basista [41] was employed. The respondent rated each of the 12 statements using a 7-point Likert scale, starting from 1 − “I strongly disagree” to 7 − “I strongly agree.” The scale considers 3 major sources of perceived social support: significant other, family, and friends. The results may be calculated both for individual subscales, as well as for the entire scale. It is assumed that the higher an individual's MSPSS score, the greater the perceived level of social support. In this study, Cronbach's α for the entire scale was 0.96, while individual subscales ranged 0.92–0.96. In turn, it was 0.86 in Polish adaptation studies [41].

So as to measure perceived stress at work, the *Perceived Stress at Work* (PSaW) scale by Chirkowska-Smolak and Grobelny [42] was applied. The tool consists of 10 items that measures unpredictability, lack of control and extensive overload by work events in the month prior to the survey (for instance, “how often in the last month have you become angry at work because you felt powerless to what happened there?”). The respondents answer on a scale from 0 − “never” to 4 − “very often.” The tool is reliable, as Cronbach's α was 0.8524 in the validation study [42] and 0.77 in this study.

The *Professional Quality of Life Scale* (ProQOL) v. 5 by Stamm [43] in a Polish adaptation by Czernecki et al. was employed to measure compassion fatigue, work satisfaction and burnout in professional helping to those that experience suffering and trauma [44]. This 30-item scale is helpful for gathering information about the mental health of a wide range of professional helpers, including those who offer emotional support and have experienced traumatic situations. The scale measures 3 aspects of life in 3 subscales: compassion satisfaction − defined as work-related pleasure and satisfaction, burn-out − understood as work-related exhaustion, frustration, anger and depression, and secondary traumatic stress − stress that emerges from work-related primary or secondary exposure to extremely stressful events at work. The respondents are asked to respond to the statements on a 5-point Likert scale from 1 − “never” to 5 − “very often.” In the study group, Cronbach's α for individual scales ranged 0.749–0.896.

The survey questionnaire concluded with personal data that was exploited to assess basic socio-demographic data (gender, age, marital status, place of residence, education, attitude toward the Catholic faith) as well as occupation-related data (years of service as a nurse, length of service in palliative care, and number of posts) using standard questions.

### Ethical consideration

The study was approved by the Witold Chodźko Institute of Rural Health in Lublin (No. 7/2023) and was conducted in accordance with the Declaration of Helsinki. All participants gave their informed consent to participate in the study. In the PAPI survey, participants signed an informed consent form in order to take part in the study. The online CAWI survey began with an electronic information form. This introductory section specified the study's purpose, the risks of participation in the study, the respondent confidentiality, the expected benefits, the voluntary nature of participation, and the right to withdraw from the study. The consent to participate in the study was confirmed by selecting “agree”, whereas selecting “disagree” redirected respondents to the survey completion.

### Statistical analysis

Continuous variables were presented as means (M) with standard deviation (SD). The Shapiro-Wilk test was used to assess conformity with a normal distribution. Categorical variables were reported as absolute numbers and percentages. Differences between groups were assessed by Mann-Whitney test or Kruskal-Wallis test with *post hoc* test. Pearson's correlation was employed to investigate the relationships between numerical variables. In addition to the aforementioned, multivariable linear regression with backward elimination (p < 0.1) was utilized to find significant predictors of total fatigue. The results of linear regression were presented as β coefficient (b) with standard error (SE). Coefficient of determination (R^2^) was applied to describe goodness-of-fit for the performed linear regression models. Statistical analyses were determined using IBM SPSS Statistics for Windows, v. 27.0 (IBM Corp., Armonk, NY, USA). P-values < 0.05 were accepted as statistically significant.

## RESULTS

### Participants' characteristics

Table 2 shows the characteristics of the study group (N = 424). The participants’ ages ranged 23–76 years, with a mean age of 50.65 years (SD = 9.99). The majority of participants were women (94.34%, N = 400) who lived in an urban area (68.4%, N = 290), and were married (68.4%, N = 290). The mean length of service in palliative and hospice care was 11.89 years (SD = 8.34), while the mean length of service as a nurse was 25.45 years (SD = 12.32).

**Table 2.** Sociodemographic and occupational characteristics of respondents – palliative care nursing, Poland, June–August 2023

Variable	Participants (N = 424) [n (%)]	M±SD
Gender		
female	400 (94.34)	
male	24 (5.66)	
Age [years]		50.65±9.99
≤39 years	66 (15.57)	
40–49 years	92 (21.7)	
50–59 years	198 (46.7)	
60–69 years	66 (15.57)	
≥70 years	2 (9.47)	
Place of residence		
village	134 (31.6)	
city	290 (64.4)	
Marital status		
married	290 (68.4)	
single	45 (10.61)	
divorced	46 (10.85)	
widow/widower	33 (7.78)	
cohabitation/informal relationship	10 (2.36)	
Relationship to the Catholic faith		
believer	363 (85.61)	
agnostic	5 (1.18)	
of different faith	10 (2.36)	
not willing to answer that question	46 (10.85)	
Education		
certified nurse	62 (14.62)	
with a specialization	73 (17.22)	
bachelor of science in nursing	53 (12.5)	
with specialization	40 (9.43)	
master of science in nursing	28 (6.6)	
with specialization	156 (36.79)	
doctor of health sciences/doctor of medicine	5 (1.18)	
other	7 (1.65)	
Experience		
overall [years]		25.45±12.32
≤9 years	61 (14.39)	
10–19 years	62 (14.62)	
20–29 years	113 (26.65)	
30–39 years	164 (38.68)	
≥40 years	24 (5.66)	
in palliative care [years]		11.89±8.34
≤5 years	125 (29.48)	
6–10 years	96 (22.64)	
11–15 years	71 (16.75)	
16–20 years	51 (12.03)	
≥21 years	81 (19.1)	
Number of workplaces		
1	250 (58.96)	
2	128 (30.19)	
≥3	17 (4.01)	
contract other than a full time job	29 (6.84)	

### Distribution of the analyzed features according to scales FAS, UWES, MSPSS, PSaW and ProQOL

Table 3 presents the results of the respondents on the scales used in the study. The total fatigue level assessed by the FAS scale was M±SD 20.78±5.41.

**Table 3.** Distribution of the analyzed features in scales – study on 424 palliative care nursing, Poland, June–August 2023

Scale	Score (M±SD)
*Fatigue Assessment Scale* (FAS) total score	20.78±5.41
*Ultrecht Work Engagement Scale* (UWES) total score	4.26±1.09
*Multidimensional Scale of Perceived Social Support* (MSPSS)	
total score	66.97±14.89
family	21.99±5.69
friends	22.11±5.67
others	22.87±5.6
*Perceived Stress at Work* (PSaW) total score	25.57±5.6
*Professional Quality of Life* (ProQOL)	
compassion satisfaction	40.59±6.67
burnout	21.14±5.56
secondary traumatic stress	23.66±5.66

### Relationship between selected sociodemographic, occupational and cognitive variables and total fatigue

Table 4 shows the relation between selected sociodemo-graphic and occupational variables and chronic fatigue. It was found that higher levels of exhaustion were found in men than women, respondents aged 50–59 years than in persons aged 40–49 years (p = 0.014), widows/widowers than in single respondents (p = 0.004), and single respondents than in those in a relationship (p = 0.003). In addition, higher levels of total fatigue were found in agnostics as compared to respondents that refused to answer questions on faith (p = 0.026), and respondents holding a doctor of health sciences/doctor of medicine degree as compared to certified nurses with a specialization (p = 0.045) and master of science in nursing (p = 0.049).

**Table 4.** Associations between selected sociodemographic and occupational variables and chronic fatigue among 424 palliative care nursing, Poland, June–August 2023

Variable	*Fatigue Assessment Scale* score (M±SD)	p
Gender		0.005^a^
female	20.54±5.20	
male	24.42±7.31	
Age		0.010^b^
≤39 years	20.29±5.59	
40–49 years	19.40±4.64	
50–59 years	21.51 ±5.28	
60–69 years	21.06±6.28	
≥70 years	17.50±2.12	
Place of residence		0.626^a^
village	20.87±5.88	
city	20.74±5.20	
Marital status		0.021^b^
married	20.40±5.30	
single	23.51±5.54	
divorced	21.20±5.91	
widow/widower	18.88±3.30	
cohabitation/informal relationship	22.50±6.74	
Relationship to the Catholic faith		0.034^b^
believer	20.85±5.63	
agnostic	23.30±1.49	
of different faith	21.60+4.10	
not willing to answer that question	19.52±3.88	
Education		0.031^b^
certified nurse	21.33±5.53	
with a specialization	20.04±5.22	
bachelor of science in nursing	20.62±5.47	
with specialization	21.38+4.28	
master of science in nursing	19.94±6.09	
with specialization	20.72±5.16	
doctor of health sciences/doctor of medicine	31.60+7.06	
other	18.86±2.36	
Experience		
overall		0.443^b^
≤9 years	20.51±5.92	
10–19 years	21.04±5.20	
20–29 years	20.40±5.46	
30–39 years	21.23±5.51	
≥40 years	19.42±3.49	
in palliative care		0.398^b^
≤5 years	20.63±5.73	
6–10 years	20.51±4.63	
11–15 years	21.86±5.66	
16–20 years	20.18±6.02	
≥21 years	20.68±5.76	
Number of workplaces 1		0.109^b^
1	21.05±5.86	
2	20.66±4.87	
≥3	21.59±4.56	
contract other than a full time job	18.50±3.47	

Statistical significance tested with:

a Mann-Whitney test;

b Kruskal-Wallistest.

Table 5 shows the relationship between total fatigue and variables assessed using standardized scales: work engagement, perceived social support, perceived stress at work and compassion fatigue. There was a significant negative relationship found between total fatigue and work engagement, perceived social support and in the “family,” “friends” and “others” categories, and professional quality of life in the “compassion satisfaction” category. On the other hand, there was a significant positive relationship between perceived fatigue and perceived stress at work, and professional quality of life in the following categories: “burnout” and “secondary traumatic stress”.

**Table 5.** Relationship between perceived fatigue (total fatigue) and variables assessed through standardized scales – Poland, June–August 2023

Variable	*Fatigue Assessment Scale* score	
	r	p
*Ultrecht Work Engagement Scale* (UWES) total score	−0.52	<0.001
*Multidimensional Scale of Perceived Social Support* (MSPSS)		
total score	−0.27	<0.001
family	−0.19	<0.001
friends	−0.25	<0.001
others	−0.28	<0.001
*Perceived Stress at Work* (PSaW) total score	0.56	<0.001
*Professional Quality of Life* (ProQOL)		
compassion satisfaction	−0.47	<0.001
burnout	0.64	<0.001
secondary traumatic stress	0.45	<0.001

### Features related to the perception of total fatigue – multivariable analysis

Table 6 shows the significant predictors of total fatigue obtained by linear regression. It was found that there was a positive relationship between gender, age, place of residence, marital status, education, the perception of social support, stress at work and professional quality of life, and the perception of the sensation of exhaustion. Higher fatigue/burnout levels were found in men as compared to women, city dwellers as compared to country residents, and persons holding a bachelor of science in nursing with specialization as compared to certified nurses. The authors also discovered that respondents who self-described having higher levels of exhaustion were those who had rating higher on the perceived social support in the “friends” than “family” category and scored higher on the burnout and secondary traumatic stress subscales, as compared to those rating higher on the compassion satisfaction subscale in terms of the professional quality of life. Furthermore, a significant negative relationship was found between the number of years of work experience, work engagement, the level of perceived social support in the “others” category, and total fatigue. Respondents that rated higher on the “others” category of social support as compared to those that rated higher on the “family” category, in contrast, experienced less fatigue (total fatigue).

**Table 6.** Relationship between perceived chronic fatigue and analyzed variables among 424 palliative care nursing, Poland, June–August 2023

Variable	b	SE	p
Gender (ref. female)			
male	0.12	0.04	0.001
Age	0.18	0.06	0.001
Place of residence (ref. village)			
city	0.13	0.04	<0.001
Marital status (ref. married)			
single	0.08	0.04	0.013
Education (ref. certified nurse)			
bachelor of science in nursing with specialization	0.13	0.04	<0.001
Years of experience	−0.12	0.05	0.023
*Ultrecht Work Engagement Scale* (UWES) total score	−0.35	0.05	<0.001
*Multidimensional Scale of Perceived Social Support* (MSPSS) (ref. family)			
friends	0.14	0.05	0.007
others	−0.14	0.05	0.005
*Perceived Stress at Work* (PSaW) total score	0.23	0.04	<0.001
*Professional Quality of Life* (ProQOL) (ref. compassion satisfaction)			
burnout	0.18	0.06	0.001
secondary traumatic stress	0.2	0.04	<0.001

R^2^ for all variables was 0.56.

## DISCUSSION

This paper assesses the level of fatigue/burnout/exhaustion among nursing staff who provide palliative care services and seeks potential predictors of this phenomenon. To the best of the authors’ knowledge, this is the first study involving such a large group of nursing staff that provides palliative care in Poland. The study's findings support the hypothesis that fatigue or burnout has multiple causes. In the study group, the level of exhaustion was largely determined by sociodemographic, occupational and cognitive variables. The findings may help to develop effective methods for proactively monitoring and managing this state in nursing staff, thereby improving healthcare quality and lowering fatigue-related risks.

High levels of burnout are reported by nursing staff as a major factor influencing career change decisions worldwide [45]. In the authors’ study, the level of exhaustion was moderate, while Jankowska-Polańska et al. [46] found that nursing staff in the Department of Paediatric Oncology, Haematology and Bone Marrow Transplantation experienced slightly lower levels of fatigue. These authors assessed fatigue levels in 95 nurses through the *Modified Fatigue Impact Scale* (MFIS). In turn, Zdanowicz et al. [47] used the FAS scale to survey 134 members of the nursing staff working in various wards and also found that respondents experienced moderate levels of exhaustion. The authors of the present paper did not find any studies that assessed the level of total fatigue among nursing staff providing palliative care services. It should be noted that current studies among palliative care nursing staff have focused on assessing compassion fatigue [48,49] which is defined as “a state of exhaustion and dysfunction-biologically, physically and socially, as a result of prolonged exposure to compassion stress and all that it evokes” [50].

The results obtained by the authors are challenging to discuss, as the understanding of fatigue or burnout assumed in the study is much broader.

The authors' study found that age, gender, marital status, place of residence, education and attitude toward the Catholic faith were the sociodemographic variables that significantly differentiated the study group's fatigue levels in both univariate and multivariate analyses. Similarly to the authors’ study, Cho et al. [51] found that there was a significant relationship between the compassion fatigue levels among palliative care nursing staff and sociodemo-graphic characteristics. Of note, Farag et al. [52] observed that female nurses experienced higher levels of work-related exhaustion. According to the authors, women are more likely to do housework when they return home from work, whereas men are more likely to rest or go to sleep. As there were significantly fewer men than women in the authors’ study and due to cultural differences in the country where the study was conducted regarding the sharing of household responsibilities, its results may differ from those in the study indicated above. The authors’ study also found that younger nurses experienced lower levels of fatigue or burnout. This may be due to their possibly higher levels of empathy and consequent acceptance of the challenging circumstances faced by the patient they were caring for, as well as differences in workload and type of work due to job seniority [53].

Greater work engagement provides psychological protection for employees as it makes them more emotionally resilient to dealing with suffering when they do not achieve therapeutic success or when the patient's health condition deteriorates [54]. The authors’ research found that as work engagement decreases, fatigue levels increase. A similar result was obtained by Cao et al. [55]. In accordance with the conservation of resources theory [56], when nursing staff experience burnout, they need resources to deal with it. In order to compensate for the loss of resources required to cope with exhaustion, nursing staff become less committed to work [57]. Consequently, work engagement is a useful metric for assessing the motivation, well-being and satisfaction with working conditions [58].

This study in univariate models found that the greater the level of support from “family,” “friends” and “others,” the lower the level of fatigue or burnout, which is consistent with the findings of Zhang et al. [59]. Nurses that are provided with support, have better physical and mental health [60]. As part of their job, palliative care nursing staff must deal with the patients’ traumatic experiences and frequently feel empathy for them. If this condition lasts too long without enough rest, nursing staff becomes fatigued. It should be noted that nursing staff provided with support from friends, family and coworkers are more likely to select active relaxation techniques that will help lower their levels of exhaustion. It is interesting to note that in the multivariate models, respondents provided with more support from friends than from family reported feeling more fatigued. On the other hand, respondents that rated the level of support in the “others” category higher than “family”, had lower levels of this. This may result from the fact that during the work shift, when the level of perceived fatigue is highest, nurses receive direct support from colleagues when dealing with challenging circumstances. As a result, managers of palliative care centers should develop appropriate habits within therapeutic teams, with the goal of encouraging all team members to actively participate in supporting one another.

In the authors' study, there was a positive relationship between stress and fatigue levels in both univariate and multivariate analyses, which is consistent with the study by Labrague et al. [61] and Lo et al. [62] An intervening variable in the relationship between perceived stress and burn-out may be the organizational culture, as demonstrated in the studies by Lee et al. [63] and Peterson et al. [64] that argue that the organizational culture influences how its members perceive stress factors, and has an impact on interactions, behavior and communication manner. Members of a relationship-oriented organizational culture with strong cooperation among individual members, or an innovation-oriented organizational culture with good communication, have lower stress levels [65]. As a result, managers of palliative care facilities must take action to create a positive workplace culture that will lower stress and fatigue levels [66] and to encourage members of the organization to support one another.

In this study, there was a negative relationship between compassion satisfaction and fatigue levels, while the “burnout” and “secondary traumatic stress” categories were positively correlated with compassion levels. In multivariate analyses, it was also found that respondents with higher scores in the “burnout” and “secondary traumatic stress” categories had higher levels of exhaustion as compared to those with higher “compassion satisfaction” scores. Factors associated with the professional quality of life of the nursing staff can be divided into situational and intrinsic. Compassion satisfaction and secondary traumatic stress are intrinsic factors, whereas burnout is a situational factor that causes the individual's gradual work-related discouragement and psychological withdrawal [67]. Poor professional quality of life among nurses is linked to fatigue levels and can lower the quality of care they provide [68,69].

The authors' work underlines the fact that the provision of palliative care to patients with advanced illnesses is a challenge not only for patients and their families, but also for medical staff. Every day, nursing staff that provide palliative care services witness patients’ pain and suffering and, as a result of their involvement in intensive care, face ethical dilemmas [69]. Close encounter with pain and suffering fosters kinship, empathy and compassion, which can lead to compassion fatigue and lower compassion satisfaction [70]. Additional factors contributing to increased occupational stress and burnout among nursing staff include the high number of patients under care, excessive workload, and a lack of free time [71]. High levels of occupational stress have a negative impact on work engagement, patient satisfaction and safety, as well as contribute to job burnout. According to Duarte et al. [72], about 25% of all oncology nursing staff members had high levels of compassion fatigue and burnout, along with low levels of compassion satisfaction, while Ja et al. [73] found high levels of secondary traumatic stress in 27.9% of the nursing staff, burnout in 35% and decreased levels of compassion satisfaction in 25.7%.

According to the authors research, nurses in Poland who provide palliative care report feeling extremely exhausted. The World Health Organization (WHO) defines fatigue as a chronic, profound and disabling state of exhaustion [9], and it is frequently caused by prolonged workload and stress, which affects nursing staff who provide end-of-life care. The study's findings are also consistent with the International Labour Organisation's (ILO) approach, which identifies excessive workload and poor quality of rest as the primary causes of fatigue [10].

At the same time, the authors' findings are consistent with the European Agency for Safety and Health at Work (EUOSHA) approach, which recognizes fatigue/burnout as a critical occupational health issue [11]. In accordance with the Scientific Committee of Occupational Health Nursing (ICOH), nurse fatigue may have a cumulative effect on the decision-making ability, physical health and cognitive function, thus influencing professional well-being and the quality of patient care [14].

The strengths and weaknesses of this study deserve consideration. Firstly, this is the first study to assess fatigue levels and their predictors among nursing staff that provide palliative care health services in Poland. Secondly, the study's findings are based on a large sample size, so they provide statistically significant information. This study also has some limitations that need to be taken into consideration. Firstly, the cross-sectional design of this study and the conducted analysis of the results limits its power to cause-and-effect inference, as it only shows a certain tendency of relationships. Secondly, the research data was collected during the summer holiday season and that could have influenced respondents’ perceptions of fatigue. Thirdly, the FAS scale only measures general fatigue levels and does not address specific types, such as compassion fatigue. Even though general fatigue and other types of exhaustion overlap, it would be beneficial in the near future to examine the specific types of fatigue experienced by Polish nurses that provide palliative care services.

## CONCLUSIONS

The study's results show a significant relationship between perceived fatigue and socio-demographic, occupational and cognitive variables. Fatigue or burnout were found to increase with age, and was more prevalent in men, those who reside in the city, and in respondents with a bachelor of science in nursing (BSN) with specialization. Additionally, respondents with more support from friends than family and higher professional quality of life scores in the “burnout” and “secondary traumatic stress” categories reported higher levels of exhaustion. However, there was a negative relationship between years of service and perceived fatigue/exhaustion. In addition, respondents that received more social support from significant others reported feeling less fatigued.

The study's findings may indicate that more funding for staff employment is required (particularly in urban facilities), in order to reduce workload and effectively manage work-related exhaustion. In addition, the study also emphasizes the significance of social support in reducing burnout among nurses. Palliative health care facilities should consider implementing structured support programs, such as peer support groups, regular debriefing sessions and psychological counselling. Out-of-work-place support networks, such as programs aimed at nurses’ families, should receive special attention. Additionally, decision-makers should prioritize programs that address healthcare workers’ overall well-being. Providing incentives to engage in fatigue reduction programs and making sure that workplace safety standards are met can bring benefits for both nurses and patients.
